# Anterolateral minithoracotomy versus median sternotomy for the surgical treatment of atrial septal defects: a meta-analysis and systematic review

**DOI:** 10.1186/s13019-021-01648-y

**Published:** 2021-09-20

**Authors:** Yu-Qing Lei, Jian-Feng Liu, Wen-Peng Xie, Zhi-Nuan Hong, Qiang Chen, Hua Cao

**Affiliations:** 1 Department of Cardiac Surgery, Fujian Branch of Shanghai Children’s Medical Center, Fuzhou, China; 2 Fujian Children’s Hospital, Fuzhou, China; 3 Fujian Maternity and Child Health Hospital, Affiliated Hospital of Fujian Medical University, Fuzhou, China; 4 Fujian Key Laboratory of Women and Children’s Critical Diseases Research, Fujian Maternity and Child Health Hospital, Fuzhou, China

**Keywords:** Anterolateral minithoracotomy, Median sternotomy, Surgery, Atrial septal defects, Meta-analysis

## Abstract

**Background:**

To compare the short-term safety and efficacy of right anterolateral minithoracotomy (ALMT) and median sternotomy (MS) for the surgical treatment of atrial septal defects (ASDs).

**Methods:**

The PubMed, EMBASE, Web of Science, and Cochrane Library databases were searched for comparative studies focusing on surgical repair of ASDs via ALMT or MS published up to the end of April 27, 2020. We used random-effect or fixed-effect models to obtain pooled estimates.

**Results:**

A total of 7 publications, including 665 patients (ALMT 296 and MS 369), were included. Age (WMD: 1.80 years, 95% CI 0.31–3.29), weight (WMD: − 0.91 kg, 95% CI − 5.57 to 3.75), sex distribution (OR: 1.00, 95% CI 0.74–1.35) and surgical type (patch or direct closure) (OR: 1.00, 95% CI 0.67–1.49) were comparable in the ALMT group and MS group. No significant differences in the success rate (OR 0.23; 95% CI 0.05–1.07) or severe complication rate (OR 1.46; 95% CI 0.41–5.22) were found between the ALMT group and the MS group. In addition, the differences in the cardiopulmonary bypass (CPB) time (WMD 6.33; 95% CI − 1.92 to 14.58 min, *p* = 0.13) and the operation time (WMD 5.23; 95% CI − 12.49 to 22.96 min, *p* = 0.56) between the ALMT group and the MS group were not statistically significant. However, the ALMT group had a significantly longer aortic cross-clamp time (2.37 min more, 95% CI 1.07–3.67 min, *p* = 0.0003). The intubation time was 1.82 h shorter (95% CI − 3.10 to − 0.55 h; *p* = 0.005), the intensive care unit (ICU) stay was 0.24 days shorter (95% CI − 0.44 to − 0.04 days; *p* = 0.02), and the postoperative hospital stay was 2.45 days shorter (95% CI − 3.01 to − 1.88 days; *p* < 0.00001) in the ALMT group than in the MS group. Furthermore, the incision length was significantly shortened by 8.97 cm in the ALMT group compared with the MS group (95% CI − 9.36 to − 8.58 cm; *p* < 0.00001).

**Conclusions:**

In the surgical treatment of ASD, ALMT and MS are equally safe and effective in terms of success rates and severe complication rates. The surgical procedures are equally difficult, but ALMT is associated with a faster functional recovery and better cosmetic results. Compared to MS, ALMT is the better choice for select ASD patients.

## Introduction

Atrial septal defects (ASDs) are one of the most common congenital heart defects, accounting for 10–15% of all forms of congenital cardiac malformations [1]. Currently, percutaneous device closure is considered the first choice for the treatment of most ASDs, with excellent outcomes and shorter hospitalization. However, surgical repair is still indicated for limited nonsecendum ASDs or secundum ASDs, characterized by large defects, insufficient rims, or a left atrium that is too small to accommodate a device [2]. Surgical repair of ASD via median sternotomy (MS) under cardiopulmonary bypass (CPB) is considered the definitive standard treatment. The mortality associated with the use of surgical treatment of ASDs is near zero. However, the use of the MS approach is limited because of the requirement for blood transfusion and the associated surgical incision scarring and prolonged recovery. Right anterolateral minithoracotomy (ALMT) has been widely applied as an alternative to MS for ASD surgical repair with similar mortality and postoperative morbidity and superior cosmetic results compared to the MS approach [3, 4]. One meta-analysis has already been conducted on ALMT versus MS for the treatment of congenital heart defects. Ding and his colleagues concluded that ALMT could benefit patients by reducing intubation time and postoperative hospital stay [5]. However, there is still a dearth of meta-analyses focusing on ALMT versus MS for ASD treatment. This study aimed to compare the short-term results between ALMT and MS for surgical repair of ASD.

## Methods

### Literature search strategy

A search of the English literature from the start date of each database up to the end of April 27, 2020, based on the PubMed, EMBASE, Web of Science, and Cochrane Library databases, was conducted by 2 independent researchers (Jian-Feng Liu and Wen-Peng Xie). The retrieval keywords included anterolateral minithoracotomy, minithoracotomy, and congenital heart defects. The search strategy was (((((anterolateral minithoracotomy[Title/Abstract]) OR submammary[Title/Abstract]) OR minimally invasive[Title/Abstract])) OR "Thoracotomy"[Mesh])) AND atrial septal defect. From this search list, studies investigating the results of the surgical treatment of ASD via ALMT or MS were identified. References of retrieved articles and reviews were also manually screened to obtain relevant eligible studies. Any disagreements were resolved through discussion or consultation with a third person.

### Study selection and quality assessment

**Fig. 1:**
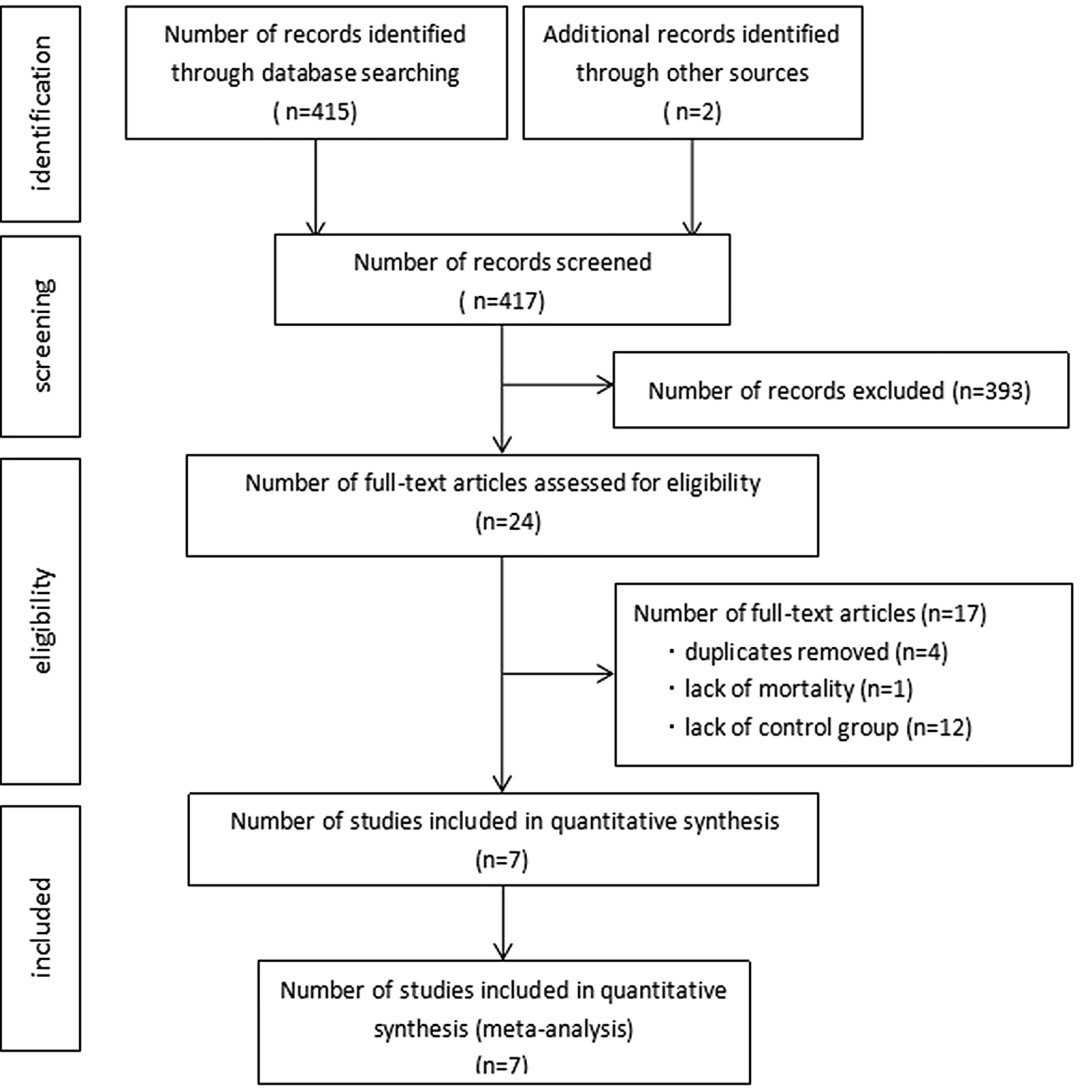
Flow chart of study selection

The inclusion criteria were comparative studies (randomized and nonrandomized studies) focusing on patients with ASD undergoing surgery via ALMT or MS. The exclusion criteria were case series already included in multicenter studies with sample sizes below 10. Our search identified 417 articles, of which 410 were excluded (Fig. 1). A total of 7 articles were finally included and further analyzed [6–12].

All 7 included studies were case–control studies, so we used the Newcastle–Ottawa Scale (NOS) to assess the quality. The NOS assesses the quality of studies based on the selection of the cases and controls (0–4 stars), the comparability of the cases and controls (0–2 stars), and the ascertainment of exposure (0–44 stars). NOS scores > 6 stars are considered to indicate high quality [13]. Disagreements in the quality assessments were resolved through discussion.

### Data extraction

The following data were extracted by two independent authors (Jian-Feng Liu and Wen-Peng Xie) and entered into an Excel sheet: publication details, first author name, sample size, patient weight, age, patient sex (male/female), success rate, operation time, aortic cross-clamp time (AACT), severe complications, intensive care unit (ICU) stay time, and the length of postoperative hospital stay. Successful closure was defined as follows: (1) no residual shunt and (2) no severe complications (including death, reoperation, neurological complications, renal failure, respiratory failure, and conversion to MS in the ALMT group).

### Statistical analysis

We used the inconsistency statistic (I^2^) to evaluate the extent of heterogeneity. An I^2^ value less than 50% indicated statistical homogeneity among studies, in which case a fixed-effect model was used. In contrast, an I^2^ value greater than 50% was considered to indicate substantial heterogeneity, in which case a random-effect model was used. A 2-sided test at the 5% level was defined as indicating statistical significance, as determined using ReviewManager (RevMan) software (version 5.4.1; The Nordic Cochrane Centre, Copenhagen, Denmark). Publication bias could not be accurately assessed following the Cochrane Handbook guidelines owing to the limited number of included studies (below 10).

## Results

A total of 7 studies (Table 1) comparing efficacy and safety in 665 patients (ALMT group: 296, MS group: 369) were included for further analysis. Age (WMD 1.80, 95% CI 0.31–3.29), weight (WMD − 0.91, 95% CI − 5.57 to 3.75), sex distribution (OR 1.00, 95% CI 0.74–1.35) and surgical type (patch or direct closure) (OR 1.00, 95% CI 0.67–1.49) were comparable in the ALMT group and MS group.

**Table 1:** Characteristic details

First author	Year	Study type	Study design	Country	RALMT (n)	MS (n)	Total pts (n)	NOS score
C H Chang	1998	Case–conrtol	RS	China	60	58	118	7
Roberto Formigari	2001	Case–conrtol	RS	Italy	71	50	121	7
Chen-hui Qiao	2003	Case–conrtol	RS	China	82	67	149	7
E Demirsoy	2004	Case–conrtol	RS	Turkey	17	36	53	8
Murat Basaran	2008	Case–conrtol	RS	Turkey	34	22	56	7
Virgilijus Tarutis	2009	Case–conrtol	RS	Lithuania	17	107	124	7
Yüksel Beşir	2019	Case–conrtol	RS	Turkey	15	29	44	8
Total pts					296	369	665	

**Fig. 2:**
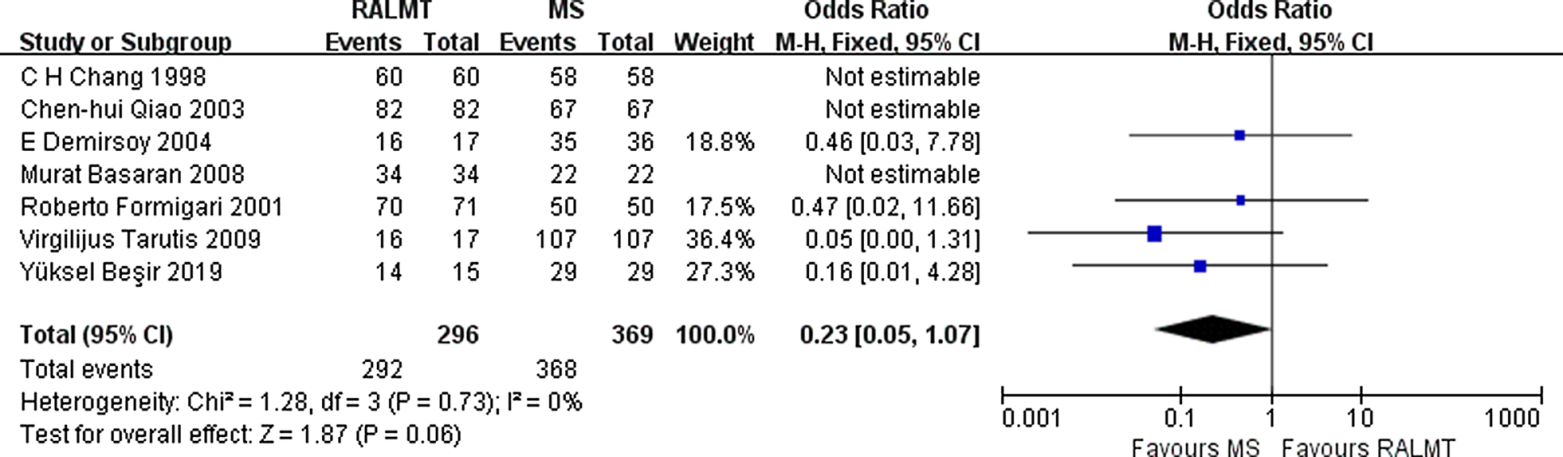
Forest plot of operational success rate

The ASD operation success rates in the ALMT group and MS group were high. The Q statistic showed no substantial heterogeneity (I^2^ = 0%, *p* = 0.73); therefore, we chose the fixed-effect model. No significant differences in success rate were found between the ALMT group and the MS group (OR 0.23; 95% CI 0.05–1.07) (Fig. 2).

**Fig. 3:**
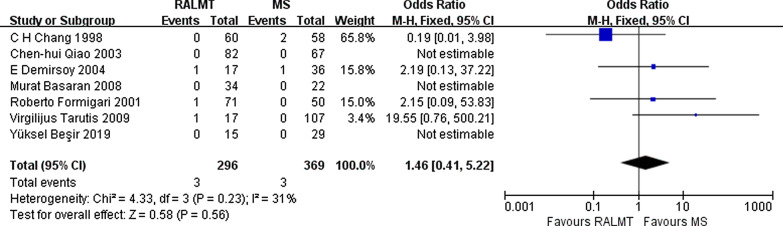
Forest plot of severe complication rate

Surgically related complications are rare in ASD surgery. Severe complications included reoperation for bleeding or severe residual disease, neurological complications, renal failure, respiratory failure, and death. The severe complication rates in the ALMT group and MS group were comparable (OR 1.46; 95% CI 0.41–5.22, *p* = 0.56) (Fig. 3). No death was reported in enrolled studies. The details of severe complications in the ALMT group and MS group are shown in Table 2.

**Table 2:** Details of severe complication in ALMT group and MS group

First author	Reoperation		Residual		Neurological complication		Renal failure		Respiratory failure	
	ALMT	MS	ALMT	MS	ALMT	MS	ALMT	MS	ALMT	MS
C H Chang	0/60	0/58	0/60	0/58	0/60	0/58	0/60	0/58	0/60	0/58
Roberto Formigari	1/71	0/50	0/71	0/50	0/71	0/50	0/71	0/50	0/71	0/50
Chen-hui Qiao	0/82	0/67	0/82	0/67	0/82	0/67	0/82	0/67	0/82	0/67
E Demirsoy	1/17	1/36	0/17	0/36	0/17	0/36	0/17	1/36	0/17	0/36
Murat Basaran	0/34	0/22	0/34	0/22	0/34	0/22	0/34	0/22	0/34	0/22
Virgilijus Tarutis	0/17	0/107	1/17	0/107	1/17	0/107	0/17	0/107	0/17	0/107
Yüksel Beşir	1/15	0/29	0/15	0/29	0/15	0/29	0/15	0/29	0/15	0/29

**Fig. 4:**
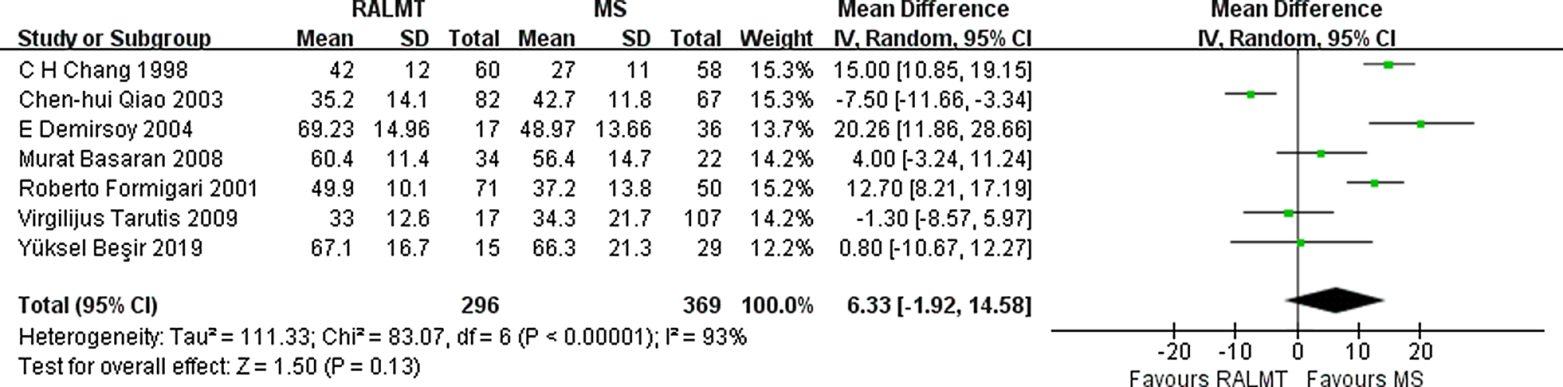
Forest plot of cardiopulmonary bypass time

The length of CPB is a useful operative measure to compare the difficulty among different types of cardiovascular surgeries. There was no significant difference in the length of CPB between the ALMT group and the MS group (WMD 6.33; 95% CI − 1.92 to 14.58, *p* = 0.13) (Fig. 4).

**Fig. 5:**
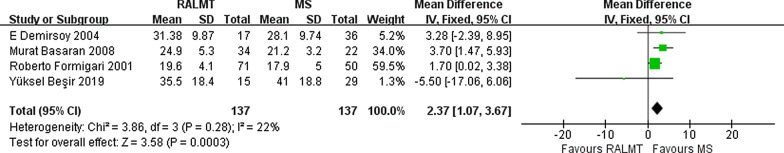
Forest plot of aortic cross-clamp time

The ACCT can also serve as an indicator of the difficulty of cardiovascular surgeries. Three of the included studies did not report ACCTs. The ALMT group had a slightly longer aortic cross-clamp time (2.37 min more, 95% CI 1.07–3.67 min, *p* = 0.0003) (Fig. 5).

**Fig. 6:**
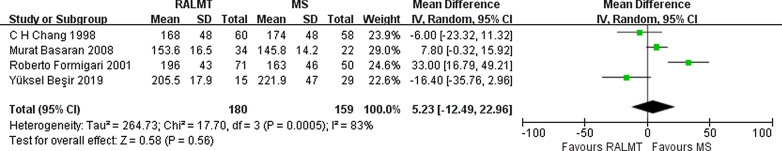
Forest plot of skin to skin operation time

The operation time can also be an indicator the difficulty of cardiovascular surgeries. We did not find a significant difference in the operation time between the ALMT group and the MS group (WMD 5.23; 95% CI − 12.49 to 22.96, *p* = 0.56) (Fig. 6).

**Fig. 7:**
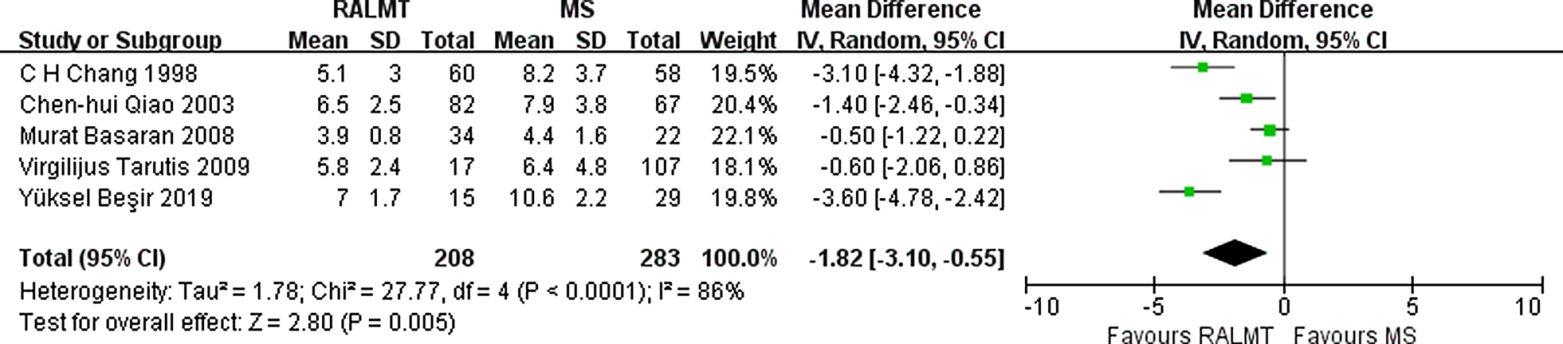
Forest plot of intubation time

The intubation time can represent the degree of lung function impairment in patients undergoing thoracotomy. The intubation time in the ALMT group was 1.82 h less than that in the MS group (95% CI − 3.10 to − 0.55 h; *p* = 0.005) (Fig. 7).

**Fig. 8:**
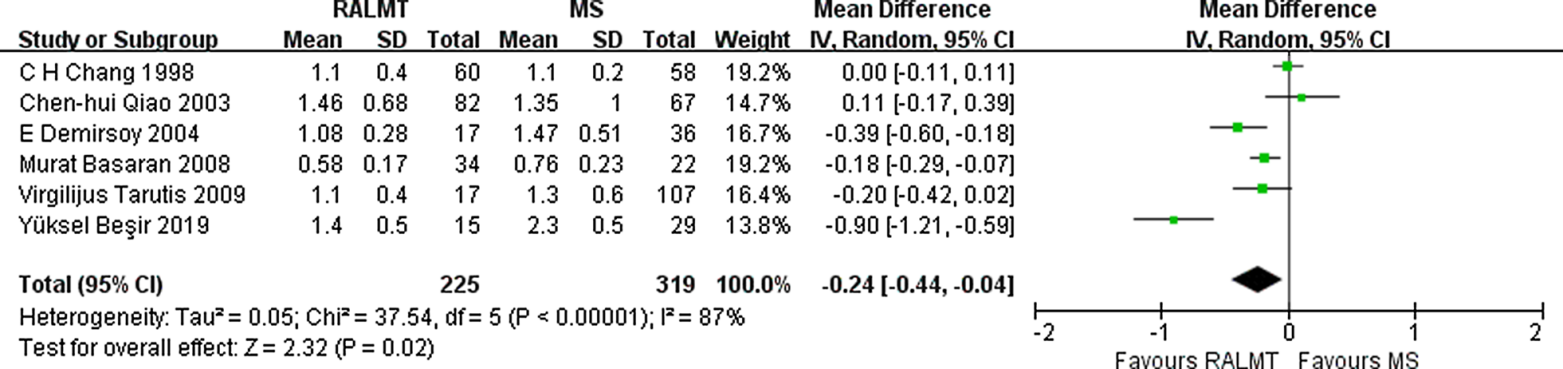
Forest plot of ICU stay time

The length of ICU stay is a sensitive indicator suggesting the recovery of postoperative patients. The length of ICU stay was significantly shortened by 0.24 days in the ALMT group compared with the MS group (95% CI − 0.44 to − 0.04 days; *p* = 0.02) (Fig. 8).

**Fig. 9:**
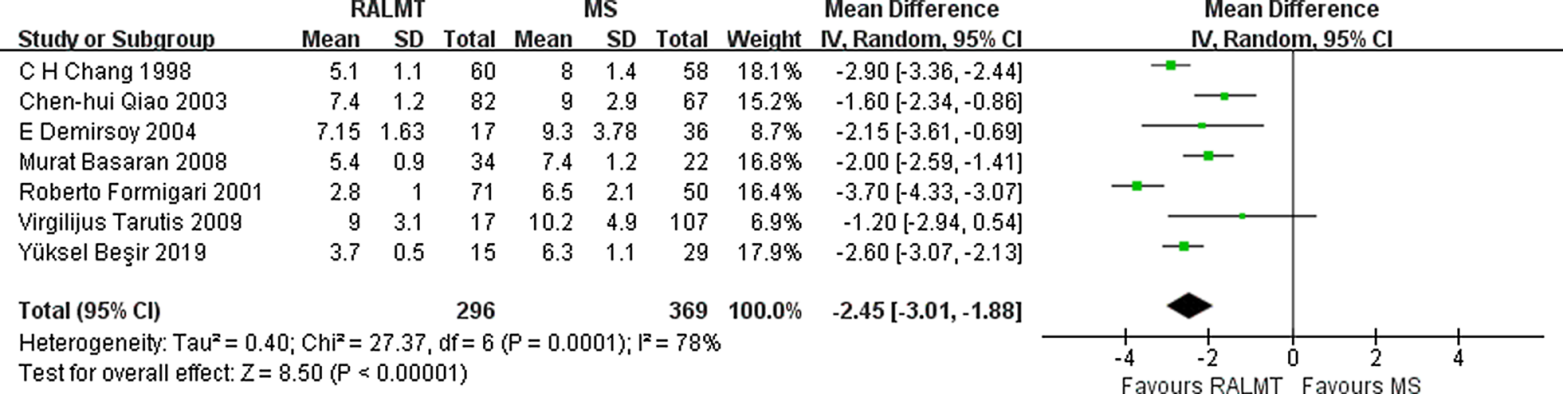
Forest plot of postoperative hospital stay time

The length of postoperative hospital stay is another outcome measure demonstrating the recovery of patients after surgery. The length of postoperative hospital stay was significantly shortened by 2.45 days in the ALMT group compared with the MS group (95% CI − 3.01 to − 1.88 days; *p* < 0.00001) (Fig. 9).

**Fig. 10:**
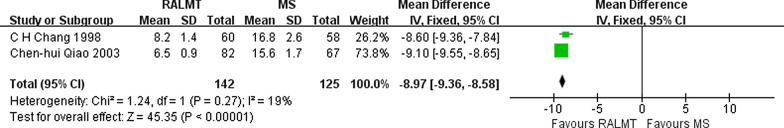
Forest plot of incision length

The incision length was significantly shortened by 8.97 cm in the ALMT group compared with the MS group (95% CI − 9.36 to − 8.58 cm; *p* < 0.00001) (Fig. 10).

## Discussion

The mortality associated with the use of surgical treatment for ASDs is near zero. The MS approach is limited because of the requirement for blood transfusion and the associated surgical incision scarring and prolonged recovery. ALMT has gained popularity for its similar mortality and postoperative morbidity and superior cosmetic results compared to the MS approach, especially for female patients.

We enrolled a total of 665 patients (ALMT 296 and MS 369) to compare the short-term safety and efficacy of ALMT and MS. No significant differences in the success rate (OR 0.23; 95% CI 0.05–1.07) or severe complication rate (OR 1.46; 95% CI 0.41–5.22) were found between the ALMT and MS groups.

In terms of cosmetic results, ALMT showed an advantage. The incision length was significantly shortened by 8.97 cm in the ALMT group compared with the MS group (95% CI − 9.36 to − 8.58 cm; *p* < 0.00001). The incisions of female patients with developed breasts could be hidden in the breast crease. Vida VL reported that 95.2% (140/147) of patients were satisfied with the cosmetic results of ALMT, with no evidence of scoliosis, asymmetric breast development, or lactation problems [14]. However, Bleiziffer and his team reported their results in a series of 71 patients (aged below 12 years) who underwent right anterolateral thoracotomy, and a breast volume difference greater than 20% (left side larger than the right) in was observed in 55% of patients, and asymmetry in the lower part of the right breast occurred in 61%. The authors recommended abandoning right anterolateral thoracotomy in prepubescent female patients, although subjective satisfaction with the cosmetic results was high [15]. Isik and his colleagues also concluded that ALMT was associated with the potential to affect unilateral breast development [16].

ALMT and MS were equally difficult to operate. There was no significant difference in the length of CPB between the ALMT group and the MS group; furthermore, the operation time was similar for the ALMT and MS groups. We argue that the two methods were near-equally complicated.

A one-lung ventilation technique, with the potential for lung injury, was applied in ALMT for ASD treatment for adequate viewing of the ASD. The ventilated lung was exposed to high strain secondary to large, nonphysiologic tidal volumes and the loss of the normal functional residual capacity. Surgical manipulation and/or the resection of the collapsed lung might induce lung injury. The reexpansion of the collapsed lung after one-lung ventilation invariably induced duration-dependent ischemia–reperfusion injury [17]. Therefore, it seemed that the intubation time should be longer in the ALMT group.

However, in cardiac surgery, lung damage is mainly ascribed to two factors: CPB and sternotomy [18]. Compared to the MS group, in the ALMT group, the intubation time was 1.82 h shorter, the length of ICU stay was 0.24 days shorter, and the length of postoperative stay was 2.45 days, but the CPB time and operation time were comparable. We attributed this difference to the fact that the ALMT group did not need sternotomy; therefore, the postoperative recovery was faster. However, the two approaches were equal in terms of the difficulty of the surgical procedure, as there was no significant difference in the CPB time or operation time. Sternotomy was burdened with postoperative complications, including mechanical impairment due to thoracic expansion and the presence of postoperative pain, which could affect breathing by decreasing the protective coughing reflex, resulting in an increase in postoperative pulmonary complications and prolonging the ICU stay and hospital stay [19, 20].

Thai published the first meta-analysis comparing ALMT versus MS for ASD treatment. The methodological strengths of the present review include (1) a comprehensive literature search following a rigorous and systematic methodology, (2) detailed data extraction, and (3) standardized quality assessment using the NOS scale.

The present review included the following methodological limitations: (1) Publication bias was not assessed according to the Cochrane Handbook guidelines in this study due to the limited number of included studies (below 10). (2) Several studies did not provide enough information. Most studies did not report minor postoperative complications, such as pain and wound infection rates, and the length of follow-up was different among the included studies. Thus, we compared only the short-term results. (3) This analysis included retrospective case–control studies but no randomized controlled studies. Thus, further studies should include a larger number of cases with sufficient data to determine the risk factors for procedure failure.

## Conclusion

ALMT and MS were equally safe and effective in ASD treatment in terms of success rates and severe complication rates. The procedures were equally difficult, while ALMT was associated with faster functional recovery and better cosmetic results. ALMT was a better choice than MS for select ASD patients.

## Data Availability

All data needed to evaluate the conclusions are present in the paper and available in the PubMed database.

## Abbreviations

ASD: atrial septal defects
CPB: cardiopulmonary bypass
ICU: intensive care unit
NOS: Newcastle–Ottawa Scale
MS: median sternotomy
ALMT: anterolateral mini-thoracotomy
RS: residual shunt
